# Effects of nicotine on markers of bone turnover in ovariectomized rats

**DOI:** 10.11604/pamj.2019.33.37.17606

**Published:** 2019-05-17

**Authors:** Panji Sananta, Andrew Jonatan, Shelby Amrus Ernanda, Ayu Novita Kartikaningtyas, Yanti Marito Parhusip, Yesi Amelia, Elli Maulidya, Muthi’ah Adira Juwono

**Affiliations:** 1 Orthopedic Department, Faculty of Medicine, Brawijaya University, Indonesia; 2 Bio Medics, Faculty of Medicine, Brawijaya University, Indonesia; 3 Faculty of Medicine, Brawijaya University, Indonesia; 4 Faculty of Medicine, Brawijaya University, Indonesia

**Keywords:** Nicotine, osteoporosis, osteocalcin, calcitonin, osteoblast, osteoclast

## Abstract

**Introduction:**

Osteoporosis is characterized by low bone mass and density, as well as change in microarchitecture of bone tissue leading to decreased bone strength. In vitro research shows nicotine can increase osteoblast activity and proliferation, also suppress osteoclast activity. Therefore we explore nicotine anti-resorptive property by in vivo true experimental and randomized posttest only controlled group research that was conducted in 18-20 weeks old *Rattus norvegicus*.

**Methods:**

Twenty-five female rats were divided into five groups, with 5 rats per group. The first group represented normal rats (Sham), while the second to fifth group underwent bilateral ovariectomy. The second group serves as positive control group (ovariectomy-only/OVX). The third to fifth group serve as dose 1 (P1-0.25mg/kg), dose 2 (P2-0.5 mg/kg), and Dose 3 (P3-0.75 mg/kg) treatment group receiving daily per-oral nicotine for 28 days, started 3 weeks post- ovariectomy. After 28 days treatment, the serum was checked.

**Results:**

Nicotine has dose-dependent manner on serum osteocalcin and serum DPD level. Level of osteocalcin in P2 group was significantly lower (Mann-Whitney, p = 0.008) compared to OVX group (59.4% lower). Level of DPD in all group was not significantly different (ANOVA, p < 0.05) but shows lowest level in P2 group. For serum calcitonin level, there's no significant different between groups.

**Conclusion:**

Nicotine at right low-dose might be able to inhibit osteoclast activity, thus open a possibility of anti-resorptive property of nicotine.

## Introduction

Osteoporosis is a disease of bone characterized by low bone mass and density, as well as change in microarchitecture of bone tissue that leads to decreased of bone strength. This disease increases the bone fragility to fracture [1]. Osteoporosis affects about 75 million people, in the United States, Europe and Japan. In worldwide, osteoporosis causes about 9 million fractures annually [2]. In osteoporosis, there is an imbalance between the activity of osteoclasts (resorption agent) and osteoblasts (bone-forming agent). This imbalance can increase bone fragility [3]. Based on in vitro research, osteoblast and osteoclast activity can be influenced by nicotine. Nicotine can increase osteoblast activity and proliferation, also suppress osteoclast activity. α4nAChR stimulation in osteoblasts can increase osteoblast proliferation rates [4]. Activation of α3 and α5nACH receptors will increase concentration of bone morphogenic protein-2 [protein with important role in bone induction regulation], and maintenance and repair during fracture healing in mouse models [5]. nAChR ligands reduced the number of osteoclasts. Stimulation of nAChR can increase osteoclast apoptosis so that it can prevent bone loss. In previous studies it was found that mice with damaged α2nAChR had osteoporosis due to up-regulation of osteoclasts [6]. Therefore, nicotine holds potency as anti-resorptive agent. The potency of nicotine as an anti-resorptive agent for osteoporosis is still poorly studied. In this study, we wanted to see the effectiveness of nicotine as a preventive agent for osteoporosis by examining bone turnover marker: serum osteocalcin, serum deoxypyridinoline (DPD) and serum calcitonin in ovariectomized wistar strain rats as model of postmenopausal osteoporosis.

## Methods

**Study design and animals:** this study was designed as in vivo true experimental and randomized posttest only controlled group research. Adult female Wistar albino rats (*Rattus norvegicus*) weighing 150-200 g and aged 18-20 weeks old were obtained from the Pharmacology Laboratory, Faculty of Medicine, Brawijaya University. Rats were housed at room temperature (27 ± 2°C) and under natural day and night cycle. They were fed standard chow pellets and drinking water ad libitum. The rats were kept for a week before the commencement of the experiment for acclimatization. The experimental protocol was approved by the Research Ethic Committee of the Faculty of Medicine, Brawijaya University (No.156/EC/KEPK/04/2017). All the experimental procedures were carried out in accordance with international guidelines for the care and use of laboratory animals.

**Nicotine preparation, dose, and administration:** we use nicotine from Sigma Aldrich with code of N0-267 100 MG. The nicotine is ethanol soluble, thus we solute it with ethanol to reach concentration of 50mg/ml. For administration, we dissolve the nicotine and ethanol solution with water to reach volume of 1 cc for each per oral administration. We use intra gastric tube to deliver the solution. The dose of nicotine was 0.25mg/kg, 0.5mg/kg, and 0.75 mg/kg of body weight per day for 28 days.

**Ovariectomy:** ovariectomy process was carried out according to protocol from physiology laboratory, faculty of medicine, Brawijaya university. Twenty-five female rats were divided into five groups, with 5 rats per group. The first group represented normal rats (sham), while the second to fifth group underwent bilateral ovariectomy. The second group serves as positive control group (ovariectomy-only/OVX). The third to fifth group serve as dose 1 (P1), dose 2 (P2), and Dose 3 (P3) respectively. Group P1 to P3 treated with the lowest to highest nicotine dose respectively. After three weeks of recovery from ovariectomy surgery, all the nicotine was administered. The first group was also administered with normal saline as placebo. The nicotine and placebo administration were given for 4 weeks.

**Animals handling and serum preparation:** at the end of the seventh week post ovariectomy, all of the experimental rats were euthanized and blood samples of each group were collected in centrifuge tubes with EDTA anticoagulant. The collected blood was centrifuged at 3000 rpm for 20 minutes and the collected sera were stored at -80°C until use.

**Bone turnover marker:** osteocalcin serum level was measured using Rat OC/BGP (osteocalcin) ELISA Kit from elabscience (E-EL-R0243). Calcitonin serum levels was measured using rat CT (calcitonin) ELISA kit from elabscience (E-EL-R0047). Deoxypiridinoline (DPD) serum levels was measured using rat DPD (deoxypyridinoline) ELISA kit from elabscience (E-EL-R0327).

**Obtaining data and analysis:** data was obtained after surgery. Statistical analysis was carried out using SPSS v. 16 software. All data were expressed as means ± standard error of mean (SEM). The data were analyzed by one-way analysis of variance (ANOVA) followed by Tukey HSD test to determine difference between groups. The data also analyzed by Kruskall-Wallis and Mann-Whitney method if the data is not suitable for parametric analysis. P values of less than 0.05 were considered statistically significant.

## Results

### Bone turnover marker levels and statistics

**Figure 1:**
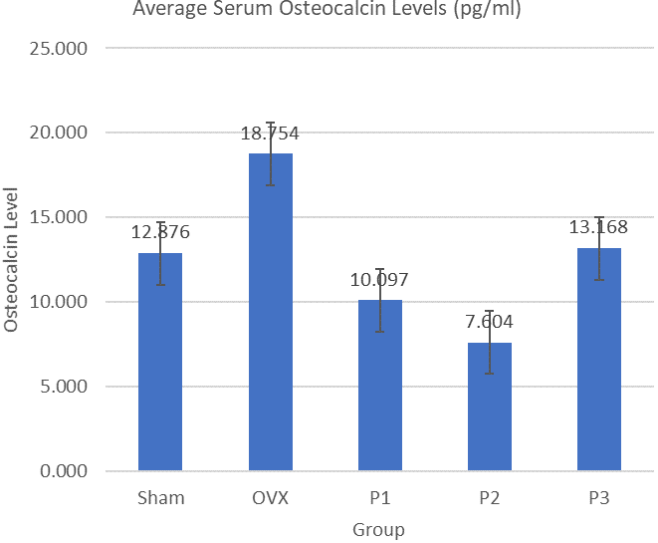
Average serum osteocalcin levels

**Figure 2:**
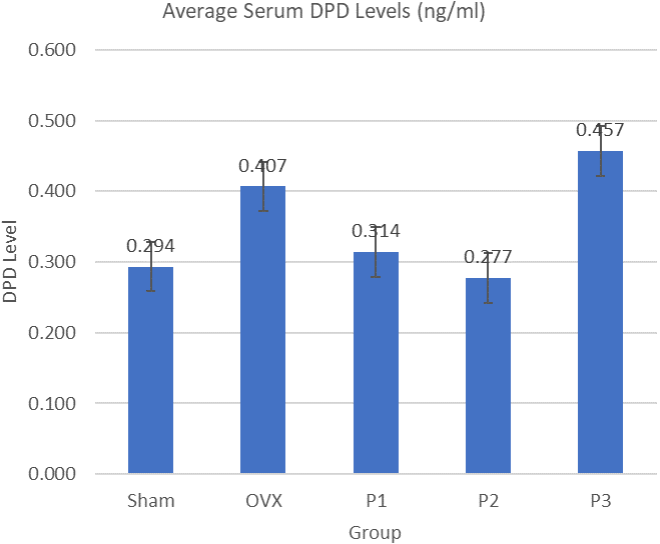
Average serum DPD levels

**Figure 3:**
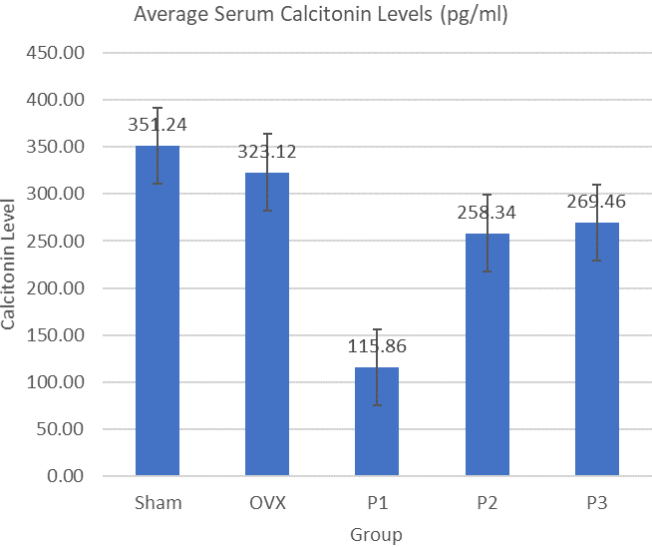
Average serum calcitonin levels

Average serum osteocalcin levels are shown in Figure 1. OVX group has 45.7% higher level of osteocalcin compared to Sham group. Level of osteocalcin in P2 Group was significantly lower (Mann-Whitney, p = 0.008) compared to OVX group (59.4% lower). The highest serum osteocalcin level was achieved by OVX group with 45.7% higher level compared to Sham group. P1, P2, and P3 have 46.1%, 59.4%, 29.7% respectively lower serum osteocalcin level than OVX group. The statistically significant decrement was achieved by P2 group (p = 0,008), and the trend suggest that nicotine administration lower the serum osteocalcin level (Figure 1). Average Serum DPD Levels are shown in Figure 2. OVX group has 38.5% higher level of DPD compared to sham group. Level of DPD in All Group was not significantly different (ANOVA, p < 0.05). P1 Group has 22.8% lower level of serum DPD compared to OVX group. The same lower results were obtained from P2 Group, where it has 31.9% lower level of serum DPD compared to OVX Group. Meanwhile P3 Group has 12.2% higher level of serum DPD compared with OVX group (Figure 2). Average serum calcitonin levels are shown in Figure 3. Sham group has 12.1% higher level compared to OVX group. Serum calcitonin level of group P1 and OVX, was significantly different (Tukey, p = 0.000). Meanwhile there's no other significant different between groups. Serum calcitonin level was highest in Sham group with 12.1% higher level compared to OVX group. All treatment groups has lower serum calcitonin level compared to OVX group, with P1, P2, and P3 have 64.1%, 20.0%, 16.6% respectively lower serum calcitonin. The statistically significant decrement was achieved by P1 Group (p = 0.000) (Figure 3).

## Discussion

Osteoporosis in rats can be observed since 2 weeks after ovariectomy [7]. It was reported that in the proximal tibia, a significant decrease in trabecular bone volume is observed 2 weeks after ovariectomy [7]. At the femoral neck, a significant decrease in trabecular bone volume occurred at 4 weeks afer ovariectomy. The lumbar vertebrae were much more resistant to ovariectomy-induced changes in trabecular bone volume than either the proximal tibia or femoral neck. It was not until 7 weeks afer ovariectomy that a decrease in trabecular bone volume was significant and reached a plateau between 39 and 77 weeks after ovariectomy [8]. In this research, we harvest the animal serum after 7 weeks post ovariectomy. Even though statistical analysis doesn't show a significant different, there was 45.7% higher serum osteocalcin levels in OVX group compared with Sham group. Osteocalcin itself is produced by osteoblasts during bone formation. Most of the osteocalcin (80-90%) is adsorbed to bone hydroxyapatite, with a minor percentage leaking into the circulation. When the bone resorption occurs, osteocalcin is released from the bone matrix into blood. Thus, serum osteocalcin concentrations increased in osteoporotic patients [9]. It was demonstrated in this research that group with daily per-oral nicotine administration has lower level of serum osteocalcin. Group P2 exhibit 59.4% lower serum osteocalcin level compared to OVX group. This phenomenon suggesting that nicotine can prevent over resorption of bone to occur. The lowered level of serum osteocalcin was relevant with findings that show stimulation of nAChR can increase osteoclast cell apoptosis so that the bone loss can be prevented. In previous study it was found that mice that were damaged in α2nAChR had osteoporosis due to up-regulation of osteoclasts [10]. It was also reported that nicotine can increases cholinergic parasympathetic nerve activity which can prevent a decrease in bone mass so that the cutting of the vagus nerve in mice causes a decrease in bone mass in the lumbar vertebra [11]. Nicotine can also reduce osteoclast activity and bone resorption by inhibiting the expression of cathepsin K, collagenase [MMP-9], vacuolar-type H + ATPase d2 and actin in osteoclast formation. Although nicotine can increase Carbonic anhydrase II, and increase activity of osteoclasts with small nuclei, nicotine also reduce tartrate resistant acid phosphatase positive multinuclear osteoclasts that have large nuclei, decrease planar areas of each osteoclast cell, decrease expression of cathepsin K, MMP-9 and V-ATPase d2, and inhibits important actin formation in podosome formation of osteoclasts [12].

Another marker of bone turnover is deoxypyridinoline (DPD) [13]. DPD is released from collagen degradation in bone resorption. It reflects the level of osteoclastic activity in the bone-remodeling process. This research shows similar trend between serum osteocalcin and serum DPD. Although statistical analysis shows the level of serum DPD in All group was not significantly different (p < 0.05), but OVX group has 38.5% higher level of DPD compared to sham group. P1 and P2 group shows lower levels of serum DPD compared to OVX group with both was 22.8% and 31.9% lower, respectively. But higher level of serum DPD was shown in P3 group compared to OVX group. It is suggesting that nicotine has dose-dependent manner as previously reported [4]. Previous study reported that only low dose of nicotine improves posterior spinal fusion in an in vivo rabbit model [14]. In menopause conditions also occur increases in the production of Reactive Oxygen Species (ROS) [15]. When ROS production exceeds the body´s capacity to produce anti-oxidants, the associated oxidative stress can cause bone damage and fragility which eventually leads to osteoporosis [16]. At low doses, nicotine can inhibit the process of lipid peroxidation caused by H_2_O_2_ and Fe_2_^+^. Nicotine inhibits the formation of H_2_O_2_ and Fe_2_^+^. Lee *et al* [17] have investigated that nicotine can induce synthesis of transcription factors NF-E2 related factor 2 [NRF2]. Nrf2 regulates the expression of HO-1. In experiments conducted on mice, administration of nicotine 1 mg/kg intraperitoneally can significantly inhibit lipid peroxidation and also increase GSH. Increased activity of HO-1 can inhibit the formation of anion superoxide and can also increase extracellular superoxide dismutase [EC-SOD], catalase and also GSH [18-20]. The present finding showed that there's no significant different between Sham group and OVX in case of serum calcitonin level. The same finding was reported by Park [21] that in ovariectomized rats there was no significantly different serum calcitonin level compared to normal rats. But the trends of calcitonin level in this research shows increased calcitonin level along with increment of nicotine dose. It shows nicotine administration with selected dose of this research failed to restore the completely calcitonin level, which, may indicate that all of selected doses were not the optimal and effective dose for restoring the calcitonin level. Thus further researches with broader dose of nicotine are needed.

## Conclusion

Based on the results of the present investigation, nicotine has dose-dependent manner on serum osteocalcin and serum DPD level. These results suggested that nicotine at right low-dose might be able to inhibit osteoclast activity. Meanwhile it was suggested that the nicotine doesn’t affect serum calcitonin levels. The possibility of osteoclast activity inhibition function of nicotine opens a possibility of anti-resorptive property of nicotine.

### What is known about this topic

- Based on in vitro research, Nicotine can increase osteoblast activity and proliferation, also suppress osteoclast activity.

### What this study adds

- We found no previous reports on nicotine effect to ovariectomized animal model;
- We wanted to see the effectiveness of nicotine in invivo studies. We used ovariectomized wistar strain rats as model of postmenopausal osteoporosis;
- We examined the effect of nicotine on bone turnover marker: serum osteocalcin, serum deoxypyridinoline (DPD) and serum calcitonin in ovariectomized wistar strain rats as model of postmenopausal osteoporosis.

## Competing interests

The authors declare no competing interests.
